# First Molecular Characterization of the Zoonotic Parasite *Balantioides coli* in *Brachyteles hypoxanthus* Wied, 1820 (Primate: Atelidae) in Brazil

**DOI:** 10.1111/jmp.70026

**Published:** 2025-06-02

**Authors:** Laís Verdan Dib, Mikaelly Frasson Testa, Fernanda Marocolo Quintão, Fernanda Pedreira Tabacow, Fabiano Rodrigues de Melo, Marcello Silva Nery, Camila Souza Carvalho Class, Alynne da Silva Barbosa

**Affiliations:** ^1^ Laboratory of Protozoology Oswaldo Cruz Institute, Oswaldo Cruz Foundation Rio de Janeiro RJ Brazil; ^2^ Campos School of Medicine (FMC) Campos dos Goytacazes RJ Brazil; ^3^ Laboratory of Parasitology, Department of Microbiology and Parasitology Biomedical Institute, Federal Fluminense University Rio de Janeiro Brazil; ^4^ Muriqui Institute for Biodiversity (MIB) Minas Gerais Brazil; ^5^ Laboratory of Virus, Department of Microbiology, Institute of Biological Sciences Federal University of Minas Gerais Minas Gerais Brazil; ^6^ Departament of Forestry Engineering Federal University of Viçosa Minas Gerais Brazil

**Keywords:** Ciliophora, gastrointestinal parasite, neotropical primate

## Abstract

Fecal samples from seven individuals kept in semi‐captivity in Minas Gerais were subjected to microscopic and molecular parasitological techniques. *Balantioides coli* was identified in all samples with a predominance of an atypical variant. This is the first molecular characterization of *B. coli* in *Brachyteles hypoxanthus* in Brazil.

## 1 Introduction

Parasitic infections in nonhuman primates (NHPs) are commonly reported worldwide and can be caused by several agents, including those considered pathogenic and with potential for zoonotic transmission, such as *Balantioides coli* [1, 2]. This parasite primarily not only infects pigs but can also cause dysentery, weight loss, and even death in humans and NHPs [3, 4, 5]. Furthermore, it has been reported to reduce fat levels in the milk of *Macaca mulatta* , potentially compromising infant development [6]. It is worth noting that the diagnosis of this parasite is often limited to microscopic detection, which can lead to misdiagnosis [7, 8]. Here, we report for the first time the molecular detection of *B. coli* in feces of *Brachyteles hypoxanthus* in Brazil.

## 2 Case Report

The study population is part of a conservation program aimed at restoring the muriqui (genus *Brachyteles*) population in the Ibitipoca region. The individuals were rescued and relocated to Muriquis House, an open‐air enclosure situated within a native forest area and dedicated exclusively to the species. The animals were introduced between 2019 and 2020, and they have been maintained under human care. As of 2024, the year in which biological sampling was conducted, the group consisted of seven individuals: three adult females, two adult males native, and two juvenile males. All sample collections were performed individually and in a manner that did not interfere with the animals' behavior. All procedures were approved by Biodiversity Authorization and Information System license number 64438‐10 and registered on the National Genetic Heritage and Associated Traditional Knowledge Management System platform by code A6EEE7D.

**FIGURE 1:**
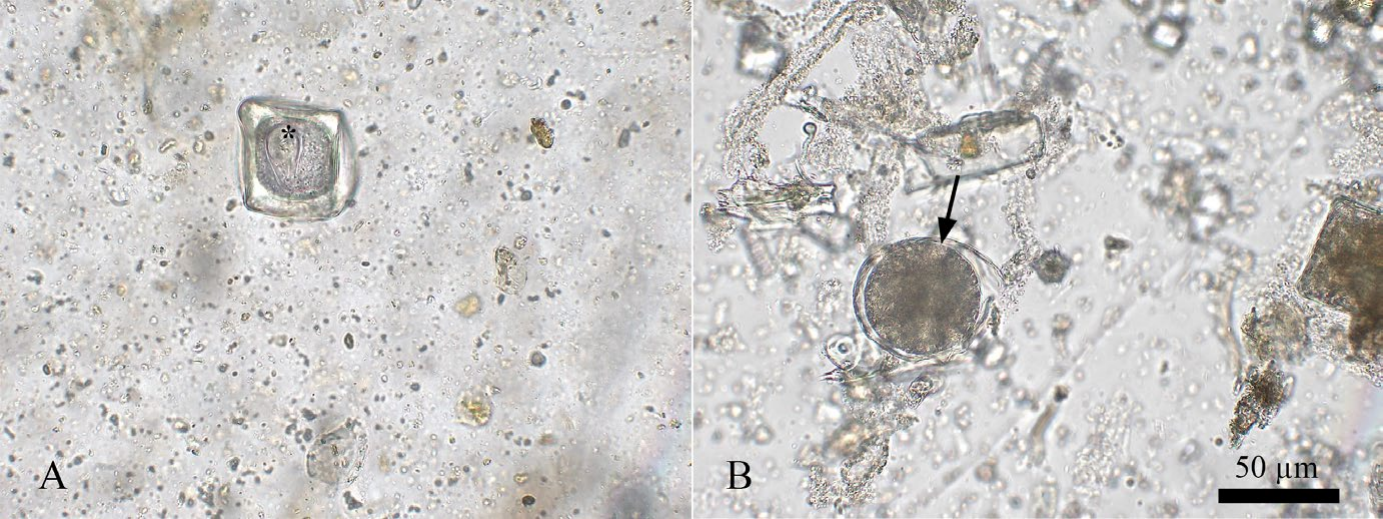
(A) Anoplocephalidae egg (*). (B) Cyst of the Ciliophora group.

Fecal collections were conducted immediately after defecation and stored in fecal collectors without preservatives. The samples were refrigerated at 4°C. The material was sent to the Parasitology Laboratory at Fluminense Federal University. The feces were processed using coproparasitological techniques, including a centrifugal flotation [9, 10], a spontaneous sedimentation [11], and a centrifugal‐sedimentation techniques [12, 13]. Microscope slide examination, morphometry, and photomicrography of parasites were conducted using an Olympus BX 41 optical microscope coupled to a BEL EU12CONVS camera with magnifications of 100× and 400×. Parasitic structures were detected in three of the seven samples; eggs of the Anoplocephalidae were identified in two samples, and a cyst of the Ciliophora Group was detected in one with a diameter of 50 μm (Figure 1).

To specifically characterize the parasite from the Ciliophora Group, DNA was extracted from all fecal samples and the negative control, which was ultrapure water, using the commercial QIAamp Fast DNA Stool Mini Kit. Polymerase chain reaction was performed with the Platinum Hot Start PCR Master Mix to amplify the ITS1–5.8S rRNA–ITS2 region, following a described protocol [14]. The amplified products were then purified using the Exo‐SAP enzyme and sequenced with a 3730 DNA Analyzer automated.

The sequence alignment was made using BioEdit software. Phylogenetic inferences were generated using maximum likelihood with the TIM2 + F+ evolutionary Model G4, with bootstrap support calculated from 1000 replicates. The optimal evolutionary model was selected based on the Akaike information criterion using W‐IQ‐Tree software. Phylogenetic tree editing and rooting were performed using MEGA‐X software. Reference sequences from *B. coli* and other ciliate species included in the analysis were obtained from GenBank.

**FIGURE 2:**
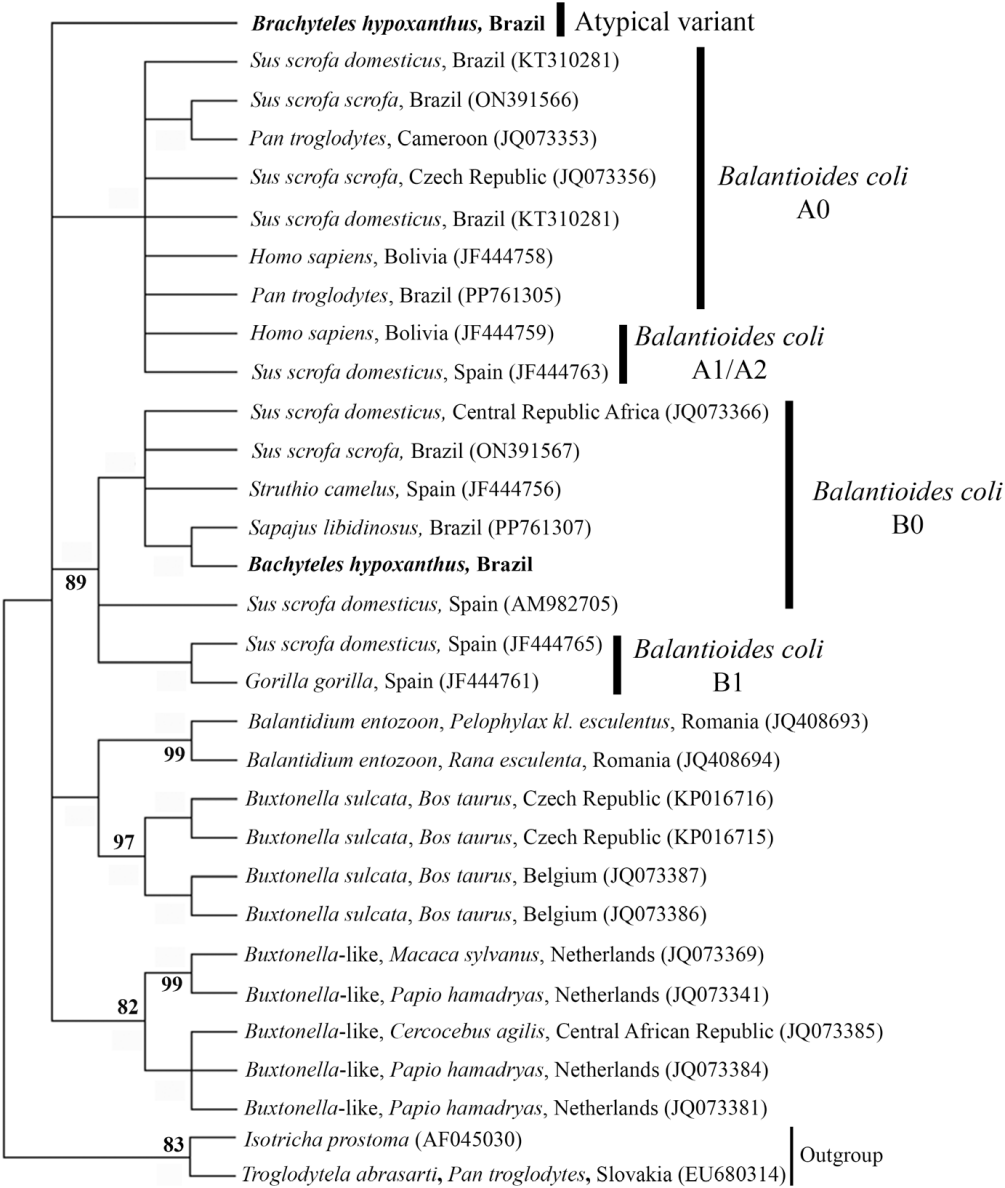
Phylogenetic tree constructed based on 370 base pairs of the DNA fragment of the ITS1‐5.8S rRNA‐ITS2 from ciliated protozoa using the Maximum Likelihood method with the TIM2 + F+ evolutionary Model G4. Bootstrap values: 1000 replications. Node numbers: Bootstrap support. Node values below 80 were omitted. Values in parentheses: GenBank accession numbers. Outgroup: *Isotricha prostoma* (AF045030) and *Troglodytela abrasarti* (EU680314).

All samples produced nucleotide sequences compatible with *B. coli*, with the B0 type variant being evidenced in the fecal material of a male muriqui. This sequence showed 99.04%–100% identity with type B0 from pigs in Brazil and the Central African Republic, ostriches from Spain, and PNH from Brazil [1, 8, 14, 15]. The six sequences obtained from the feces of other individuals did not exhibit a defined variant pattern. However, they showed the greatest similarity with type A sequences, with identities ranging from 93.9% to 94.9%. These sequences were most like those from pigs in Brazil, Spain, the Central African Republic, and the Czech Republic [8, 14, 15, 16], NHP in Brazil, Spain, and Cameroon [1, 8, 14], humans in Bolivia, and ostriches from Spain [14] (Figure 2). All sequences were deposited in Genbank with accession numbers PQ877726—PQ877732.

## 3 Discussion

This case represents the first characterization and molecular confirmation of *B. coli* in a *Brachyteles* group kept under human care in Brazil. The diagnosis of this parasite in *B. hypoxanthus* in Brazil had only been reported through microscopic methods, as observed in Espírito Santo [17], and in Minas Gerais [18]. Molecular techniques were essential to demonstrate exposure to *B. coli*, since in most samples no parasitic forms were visualized under microscopy. Furthermore, molecular techniques, despite being more expensive, provide far more accurate results for species characterization within the Ciliophora group, as other ciliated protists with highly similar morphology can also infect primates.

The remaining sequences, while confirmed as *B. coli*, displayed an atypical pattern with multiple nucleotide insertions characteristic of both variants A and B. The detection of a variant may be related to the predominance in the samples of parasite DNA sequences that present type‐specific nucleotide variations. This difference may be related to the unequal amount of genetic material in the parasite macronucleus [19]. In the case of *B. coli*, A0 and B0 sequences with high identity are diagnosed in different animal species. This fact highlights the low specificity of the parasite per host, which favors its transmission.

None of the individuals exhibited clinical signs consistent with balantidiasis. The origin of these infections is still unknown, but they may have been acquired from the ingestion of cysts in water, leafy greens, and fruits contaminated with the feces of other animals. A treatment protocol using ivermectin and praziquantel biscuits was administered. However, these medications are not effective in eliminating *B. coli*. The presence of pigs in the peridomicile of Muriqui House must be avoided to prevent new infections.

## Conflicts of Interest

The authors declare no conflicts of interest.

## Data Availability

The data that support the findings of this study are available from the corresponding author upon reasonable request.
